# Evaluation of empathy and fatigue among physicians and surgeons in tertiary care hospitals of Rawalpindi

**DOI:** 10.12669/pjms.37.3.1973

**Published:** 2021

**Authors:** Zubaida Rashid, Imtenan Sharif, Imdad Ali Khushk, Abdullah Anis Raja

**Affiliations:** 1 Zubaida Rashid, Community Medicine Department, AMC, National University of Medical Sciences, Rawalpindi, Pakistan; 2 Imtenan Sharif, Community Medicine Department, AMC, National University of Medical Sciences, Rawalpindi, Pakistan; 3 Imdad Ali Khushk, Pakistan Medical and Dental Council, Islamabad, Pakistan; 4 Abdullah Raja, Community Medicine Department, AMC, National University of Medical Sciences, Rawalpindi, Pakistan

**Keywords:** Empathy, Fatigue, Physicians, Surgeons

## Abstract

**Background and Objectives:**

Clinicians need to build an astute doctor-patient relationship. The term clinical empathy is the ability of doctor to cognitively appreciate a patient’s perspective, experiences, and deliver such an understanding back to patient. Studies associate high physician empathy with improved patient trust and clinical outcomes. In Pakistan, there is paucity of research data on this relationship. This study assessed the empathy and its relation to fatigue levels among physicians and surgeons.

**Methods:**

This was a cross-sectional analytical study involving 262 Physicians and Surgeons (1:1) chosen by convenience sampling method. Physicians and Surgeons were included from three hospitals in Rawalpindi from September 2017 to February 2018 and RAO soft sample calculator was used. The Jefferson Scale for Physician empathy (JSPE) (score range 20-140) and Multidimensional Fatigue Inventory (MFI-20) (score range 20-100) were used as data collection tools. The data was analyzed using SPSS version 23. Statistical tests including T-test and Pearson Correlation were used.

**Results:**

Overall, mean score of empathy was found to be 98.8±21.9 (range; 46-138). The empathy in Physicians (106.8±18.3) was found to be greater than Surgeons (89.4±22.1) and the difference was statistically significant (p value <0.01). Mean score of fatigue was 50.6±16.0. The fatigue level in Surgeons was greater than Physicians and the difference was found to be statistically significant (p value < 0.01). Strong negative correlation was observed between empathy and fatigue (r= -0.5, p=<0.01) using Pearson correlation.

**Conclusion:**

Overall, physicians had better empathy than surgeons, while fatigue scores were higher among surgeons. Fatigue is associated with empathy decay. This research provides an understanding of empathy deterioration and other factors responsible for it.

## INTRODUCTION

The concept of empathy is intricate, empathy has been considered a vital tool for integrating different cognitive and emotional factors.^1^ Empathy, a core element of medical practice is imperative for establishing interpersonal relationship.^2^ Physician empathy is the ability to cognitively appreciate a patient’s perspective and experience and deliver such an understanding back to patient.^3^ Historically, empathetic physician interaction with the patient has been recognized as an important factor for effective medical care. There is now an increasing agreement on the opinion that empathy is not a single ability but a “complex socio-emotional” competency that covers different interacting components. Spiro reasoned empathy as foundation of patient care. Patient physician communication is a basic medical activity and the success of this interaction is dependent upon physician’s empathy.^4^,^5^

Several factors interplay in influencing empathy during education and training in medical profession which include, years of experience, type of specialty, type of personality, gender, and cultural differences.^6^ A research on medical students and residents comparing their empathy levels (based on the Jefferson Scale of Empathy) according to years of education and training showed that empathy decreased with increasing years of experience.^6^

Empathy is also associated with physician’s well-being and reduced symptoms of fatigue. Long working hours, increase in stress, lack of sleep and decrease in leisure time lead to increased risk of developing fatigue.^7^ The number of continuous duty and work hours for medical personnel are much greater makes public safety directly at risk.

In literature, fatigue has been considered as a major factor which influences medical personnel performance and its elimination can improve medical practice, efforts to reduce fatigue should be considered responsibility of the current system.^8^ Despite an extensive volume of research data in fatigue and performance, information relating directly to physicians is inconsistent.

It is acknowledged by medical personnel that fatigue contributes to adverse patient outcomes.^9^ Although the issue of increased workload declining medical professional’s empathy is known but not enough efforts have been made to address this issue. Appropriate levels of clinical empathy can be difficult to maintain with the increased levels of fatigue.^10^ Increasing depersonalization and emotional exhaustion, because of burnout, negatively impacts physicians’ empathy.^11^,^12^ The lack of an unanimously definite operational definition of empathy ceases to exist as many schools of thought have interpreted it in their own ways. This is the reason for the scarcity of work done in this domain and consequently its effect on doctors.^13^ Our study aims to evaluate empathy as well as fatigue levels among physicians and surgeons and to determine association between levels of fatigue and clinical empathy.

## METHODS

### Study design and participants:

This cross-sectional analytical study was conducted at Military Hospital Rawalpindi, Combined Military Hospital Rawalpindi and Fauji Foundation Hospital from September 2017 to March 2018. The study recruited 262 Physicians and Surgeons (1:1). Sampling technique was done through convenience sampling. Consultants and post graduate trainees who had at least three years of professional experience were included in the study. An online sample size calculator^11^ was used with 95% confidence interval and 5% margin of error for sample size calculation which yielded a sample size of 262 healthcare professionals.

### Evaluation Tools:

The Jefferson Scale for Physician empathy (JSPE) (score range 20-140) was used for measuring empathy. The version of JSPE selected was the Health Profession (HP) version which contains 20 Likert scale items (7-point scale) to evaluate the empathy levels among the health care professionals. The scoring was done from a total of 140 points by summating the score of each question.^14^

Multidimensional Fatigue index (MFI-20) (score range 20-100) was used for measuring fatigue. This inventory is a 20-item (5-point scale) self-report validated instrument designed to measure fatigue. Its five domains include: General Fatigue, Physical Fatigue, Mental Fatigue, Reduced Motivation and Reduced Activity. Scoring is done by adding each individual score (100 being the highest score).

### Data Analysis:

A higher score on both scales indicates higher levels of Empathy and Fatigue. The data was analyzed using SPSS version 23 and Microsoft Excel. Statistical tests including T-test and Pearson Correlation were used. Level of significance was set at p≤ 0.05 to be considered statistically significant.

### Ethical Statement:

This study was approved by the institutional review board of National University of Medical Sciences, Pakistan on October 19, 2018. Written informed consent was taken from all study participants before commencement of research.

## RESULTS

This data was collected from 262 health care professionals which included 101(38.5%) consultants and 161(61.5%) post graduate trainees working in outpatient and inpatient departments. Male to female ratio of 2:1 with 166 (63.4%) males and 96 (36.6%) females respectively. The health care professionals were further stratified into 2 subcategories comprising for 142 (54.2%) physicians and 120 (45.8%) surgeons.

### Empathy Scores:

**Fig.1:**
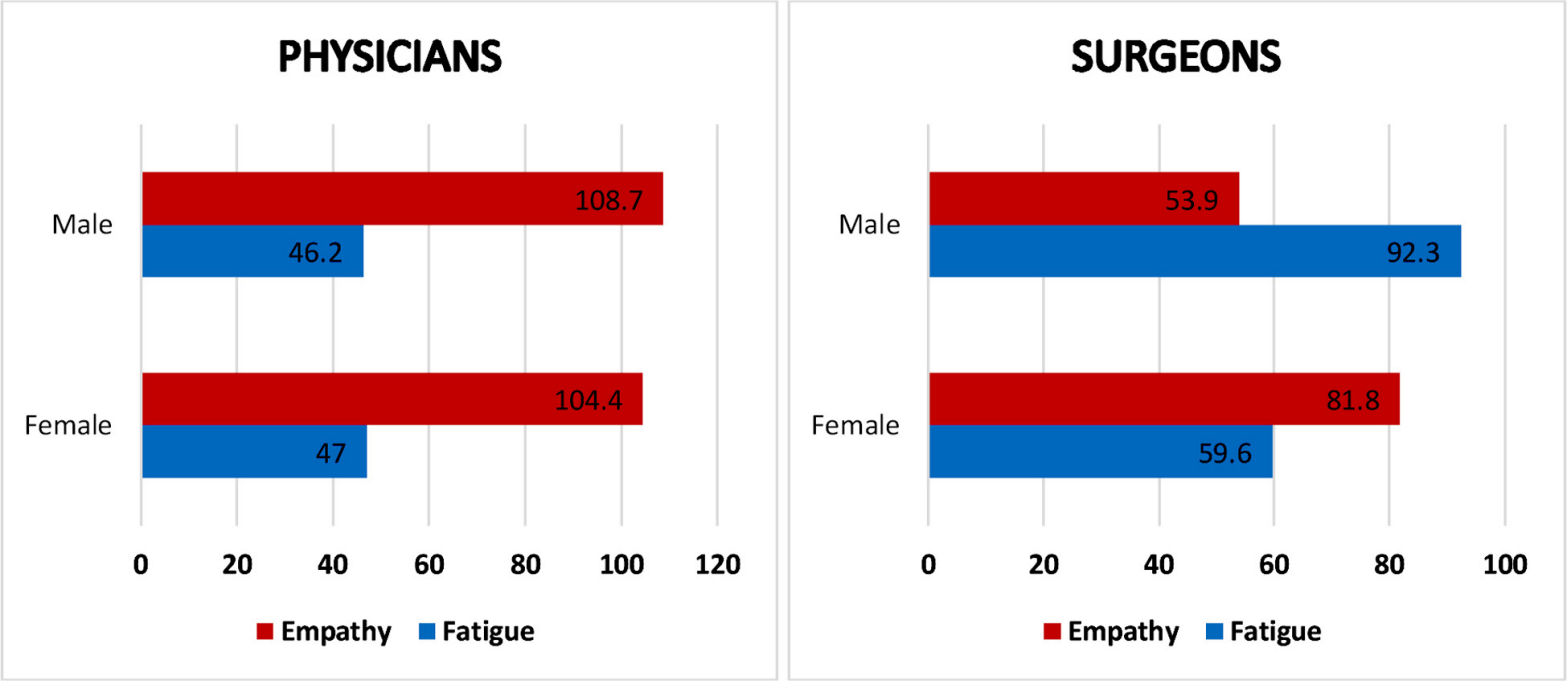
Mean score of empathy and fatigue among physicians and surgeons with respect to gender.

The overall mean score of empathy measured by Jefferson’s scale was 98.84±21.85 from a total of 140. The empathy scores in males and females were 100.1±21.59 and 96.66±22.25 respectively with no statistical significance. Empathy scores in physicians (106.81±18.27) were found to be statistically better (P value < 0.001) than that of the surgeons (89.4±22.05) (Fig.1). There was also no significant difference between the mean empathy scores of consultants (99.15±23.1) and post graduate trainees (98.64±21.1).

### Fatigue Scores:

**Fig.2:**
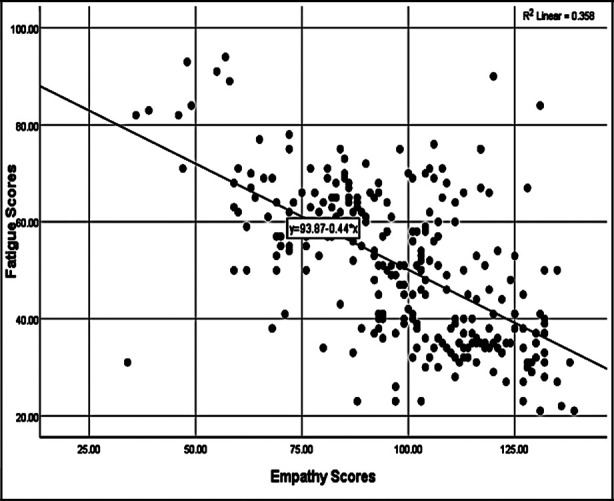
Association between empathy and fatigue.

The overall mean fatigue score of our participants was 50.62±15.97 from a total of 100. Overall, the mean fatigue scores among males and females were 50.21±16.47 and 51.34±15.12 respectively with no statistical significance. However, when analyzed according to specialties, there was a statistical significant difference in empathy scores of physicians (46.6±14.5) and surgeons (55.4±16.3) was observed (p<0.001) noting that surgical specialty had higher fatigue levels when compared with medicine (Fig.2). There was however, no statistical difference between the fatigue scores of consultants (48.01±16.26) and trainees (52.26±15.62). Comparison of empathy and fatigue levels reflected that the empathy levels were higher in physicians (106.81±18.27) than in surgeons (89.4±22.1) while the fatigue score was higher among surgeons (55.4±16.35) when compared with physicians (46.6±14.5).

Male physicians had a greater mean level of empathy (108.7) and a lower mean fatigue level (46.2) than female physicians (Fig.1). Male surgeons have a lower mean level of empathy (53.9) and greater mean fatigue level (92.3) than females of the same specialty (empathy 81.8, fatigue 59.6).

The association between empathy (measured by Jefferson’s scale of physician empathy) and fatigue (measured by MFI-20) was analyzed in the complete study group and a statistically significant, strong negative correlation was observed between empathy and fatigue (r=-0.5, p=<0.01) using Pearson correlation (Fig.2).

## DISCUSSION

Overall, the results reflect that physicians had a higher level of empathy when compared to their surgical counterparts. This result was supported by a similar study carried out in Poland in which also non-surgical specialists showed a higher level of empathy than surgical specialists.^13^ Similarly, in a study carried out in USA, the mean empathy score among physicians using Jefferson scale of empathy came out to be 120 ± 12 as compared to 106.8 ± 18.3 in our study.^14^ The predictability of this result can be supported by the finding that even in medical school; students that aim to pursue medical specialties show a relatively higher level of empathy as compared to their future surgical counterparts, which is later reflected in practice.^15^ Moreover among surgeons, empathy has an indirect effect on patient as interaction with surgeon is more during the post-operative period.^16^ Higher levels of empathy benefit the patients as a study has shown that patients of physicians with higher empathy scored had a significantly lower rate of acute metabolic complications.^17^ Our study demonstrated no significant difference between empathy scores among gender which might be due to lower study sample and sampling technique which was in contrast with other studies.^18^,^19^

Fatigue not only leads to treating patients in a depersonalized manner but also has adverse effects on the health of Doctors themselves.^13^,^20^ High levels of fatigue and low levels of empathy lead to deterioration of clinical outcomes.^21^ Fatigue was evaluated in five dimensions; mental fatigue, physical fatigue, general fatigue, emotional fatigue and loss of motivation. Studies have shown that high levels of fatigue have contributed to increased incidence of self-perceived medical errors.^22^ Surgeons showed a higher level of fatigue as compared to the non-surgical Physicians. This may be ascribed to the extensive physical nature of their work.

Highlighting the gender sway, our research found fascinating results. Though overall, there was no significant difference in empathy and fatigue scores among gender, however, this result was supported by a study held in Pakistan which calculated the empathy levels using Jefferson scale of empathy.^23^ However among physicians, female Physicians had lower empathy levels as compared to male Physicians. This result contrasted with the study conducted in Japan, which also used Jefferson scale of empathy and reflected that the levels of empathy among female Physicians had increased rapidly in the past ten years.^24^ This contrast may be due to higher levels of stress and social responsibilities on women in our setup. They are more likely to experience work home conflicts and show symptoms of depression.^25^,^26^ The fatigue levels among female surgeons were less when compared to male surgeons. This result contrasted to a study done in America which showed significantly higher fatigue levels among female surgeons.^26^

The fatigue levels among female physicians were higher as compared to their male counterparts. This result is supported by another study conducted in USA which stated higher fatigue levels among female physicians.^26^ Overall, the whole studied group showed a significant but inverse relationship (p < 0.01, R= -0.5) between empathy and fatigue levels. The same inverse correlation was observed between empathy and fatigue in a study conducted on physicians and surgeons in Poland.^13^ Further studies are required to explore the influence of gender on empathy and fatigue among Asians, and why the levels differ with respect to specialties.

## CONCLUSIONS

This study shows that there is low level of empathy in Physicians and Surgeons. Among the two, surgeons had lowest empathy scores. Fatigue levels were found to be high in surgeons as compared to the physicians. Negative correlation was found between levels of empathy and fatigue in the whole group. Male Physicians had greater level of empathy and lower levels of fatigue than females while male surgeons were found to have lower level of empathy and greater levels of fatigue than female surgeons. It is important to understand that all those factors that increase fatigue negatively impact on the empathy of the doctor. This study provides an understanding of empathy deterioration and other factors responsible may be researched to better medical practice.

### Authors Contribution:

**ZR, IS, IAK:** Conceived, designed and did statistical analysis & editing of manuscript, is responsible for integrity of research.

**AR:** Did data collection and manuscript writing.

**ZR:** Did review and final approval of manuscript.
